# Dynamical entropic measure of nonclassicality of phase-dependent family of Schrödinger cat states

**DOI:** 10.1038/s41598-023-43421-2

**Published:** 2023-09-27

**Authors:** M. Kalka, B. J. Spisak, D. Woźniak, M. Wołoszyn, D. Kołaczek

**Affiliations:** 1 Faculty of Physics and Applied Computer Science, AGH University of Krakow, al. Mickiewicza 30, 30-059 Krakow, Poland; 2 Department of Applied Mathematics, University of Agriculture in Kraków, ul. Balicka 253c, 30-198 Kraków, Poland

**Keywords:** Quantum mechanics, Theoretical physics

## Abstract

The phase-space approach based on the Wigner distribution function is used to study the quantum dynamics of the three families of the Schrödinger cat states identified as the even, odd, and Yurke–Stoler states. The considered states are formed by the superposition of two Gaussian wave packets localized on opposite sides of a smooth barrier in a dispersive medium and moving towards each other. The process generated by this dynamics is analyzed regarding the influence of the barrier parameters on the nonclassical properties of these states in the phase space below and above the barrier regime. The performed analysis employs entropic measure resulting from the Wigner–Rényi entropy for the fixed Rényi index. The universal relation of this entropy for the Rényi index equal one half with the nonclassicality parameter understood as a measure of the negative part of the Wigner distribution function is proved. This relation is confirmed in the series of numerical simulations for the considered states. Furthermore, the obtained results allowed the determination of the lower bound of the Wigner–Rényi entropy for the Rényi index greater than or equal to one half.

## Introduction

The application of space-phase methods to the study of quantum systems initiated by Wigner allows looking at the theory of these systems as the statistical theory in which observables characterizing them form a non-commutative algebra^1–3^. This observation is the cornerstone of the space-phase formulation of quantum theory and has given the impulse to develop more rigorous rudiments of this approach^4–8^. As a result of these studies, quantum mechanics emerged as a deformation of the symplectic structures characteristic of classical mechanics formulated in the phase-space language. Hence, the description of quantum phenomena is based on ordinary c-number functions on the phase space, and Planck’s constant is treated as a measure of a Poisson algebra deformation^9^. Historically, the first formulation of quantum mechanics in this way is based on the Wigner distribution function (WDF), which plays the role of the quantum system state within this approach^1^. Since then, this approach has attracted a lot of attention because of its applicability in many modern quantum problems, including quantum entanglement^10,11^, quantum computing^12^, or quantum metrology^13^. In addition, numerous applications can also be found in quantum optics^14^, atomic physics^15,16^, electrodynamics^17^, and plasma physics^18^, condensed matter^19,20^, gravitation and cosmology^21–23^ or field theory^24^. Furthermore, this approach has many interdisciplinary applications^25^, e.g. in quantum electronics^26,27^, quantum chemistry^28^, quantum biology^29,30^, or signal processing^31–33^. Besides that, Wigner’s idea is also influential in developing some branches of mathematics, e.g. non-commutative geometry^34,35^, geometrical quantization^36^, or the theory of pseudo-differential operators^37,38^.

One of the characteristic properties of the WDF is its negativity in some regions of the phase space. This property renders the WDF an example of a non-classical distribution function since the rest of this function’s properties are consistent with its Kolmogorov counterpart in probability theory. The negativity of the WDF has been the subject of numerous discussions and interpretations. Among these various proposals, the approach assuming that the WDF is treated as a wave function defined on the phase space deserves special attention^39–41^. The immediate consequence of this approach is an interpretation of this function as the probability amplitude on the phase space. Thus, the square of the absolute value of this wave function is treated as the probability density on the phase space, and just like the WDF this quantity is symplectically covariant^42^. However, this probabilistic interpretation is restricted to pure states only. Nevertheless, let us note that this new look at the WDF is free of its sign problem. On the other hand, numerous studies on the negativity of the WDF led to the conclusion that this part of the WDF is a hallmark of the state nonclassicality which can be expressed quantitatively by the nonclassicality parameter introduced by Kenfack and Życzkowski^43^. Of course, this measure is not perfect because it does not detect nonclassical states described by the WDF-positive states. Nevertheless, the advantage of the aforementioned nonclassicality parameter is its simplicity and clear interpretation because it provides information on the fraction of the phase space occupied by the negative part of the WDF.

Let us note that looking at the phase-space formulation of quanta as a statistical theory immediately inclines one to introduce the concept of entropy to the considerations as an essential component of such a statistical approach. However, at the beginning of the deliberations on the introduction of this state function, we encountered fundamental conceptual difficulties related to the negativity of the WDF. One of the first attempts to define the entropy of quantum states using the WDF has been based on the concept of coherent states, regarded as the most classical quantum states^44^. Such states are usually represented by the Gaussian functions, which minimize the uncertainty principle, and the corresponding WDF being also a Gaussian is always nonnegative, according to Hudson’s theorem^45^. In this case, considering the concept of a quantum state in the definition of entropy seems fully justified. A major step in the development of the concept of quantum state entropy in the phase-space approach based on WDF was also the work of Manfredi and Feix^46^, in which the authors introduced and discussed properties of the so-called quantum linear entropy, utilizing for this purpose the functional of the WDF square being an invariant quantity under symplectic transformation. Recently, a slightly different approach was proposed by van Herstraeten and Cerf^47^. These authors introduced the concept of Wigner quantum entropy based on quantum states with the positive definite WDF. In turn, their studies have been generalized to the case of arbitrary absolutely integrable Wigner functions by Dias et al.^48^ The results presented in these last works partially motivate our studies because their authors operated with the WDF interpreted as the wave function on the phase space. Another way to introduce the entropy within the phase-space approach for quantum states requires projecting a quantum state on the coherent state and then taking the square of the absolute value of this function, which leads to another phase-space quasi-distribution function called the Husimi distribution function^49^. This approach brought Wehrl to the concept of the von Neumann entropy in the phase space, which is currently regarded as the quantity which is the closest to the classical understanding of the entropy. In turn, the Wehrl result can be regarded as a particular case of the Rényi–Wehrl entropies^50^ commonly used to measure quantum state localization in the phase space. It is also worth to mention the Tsallis entropy, which can be seen as a linearization of the Rényi entropies. This observation was used by Sadeghi et al.^51^ to conclude that the nonclassicality parameter is related to the Tsallis entropy based on the Wigner function with the Tsallis index equal one. However, these authors did not give any quantitative characteristics in the form of a formula linking these two quantities. Instead of this, they examined several examples of states and showed the coincidence of the nonclassicality parameter and the Tsallis entropy of the Wigner function in graphs. As a result, this allowed them to conclude that these two considered quantities have similar properties. Following these results, we have found the exact relation between the nonclassicality parameter and Wigner–Rényi entropic measure of $|\rho (x,p,t)|^2$ for a fixed, fractional Rényi index value. Therefore, we can interpret the Wigner–Rényi entropy for this fractional index in terms of the nonclassicality parameter. A detailed analysis of this issue is one of the subjects which we consider in this paper.

Another issue discussed in this paper concerns the interaction of the phase-dependent family of the Schrödinger cat (SC) states with the potential barrier in a one-dimensional dispersive channel. This continuation of our previous studies on the SC states in the phase-space formalism^52^ allows us to generalize them to the case of the well-separated bimodal state formed by the coherent superposition of two Gaussian wavepackets, each of which moves with an opposite momentum value. It means that each wave packet that is a part of this coherent superposition approaches the other in the configurational space, with an obstacle in the form of a Gaussian barrier between them acting as a scattering centre. For the described situation, we performed studies in the above- and below-barrier regimes, simultaneously analyzing the influence of the relative phase encoded in these states on the nonclassicality, which is expressed in terms of the appropriate entropy. Such framework can serve as a model for scattering in the constricted semiconductor nanowires^53^. On the other hand, the process of double-sided barrier penetration has recently gained increased interest with its use in the field of quantum electron optics^54–59^.

In this work, we use three families of the SC states, i.e. even SC states, odd SC states, and Yurke-Stoler (YS) states. All of them have been extensively studied in quantum optics^60^, including quantum spectroscopy methods^61,62^, quantum computing and information theory^63^, and even quantum theory of gravity^64^. It is also worth mentioning that some experimental methods exist for creating these states. Among them, we can note intense laser-matter interaction^65^, diamond mechanical resonations^66^, levitating ferromagnetic particles^67^ or following the original Yurke and Stoller proposal with a nonlinear Kerr interaction in the superconducting circuit^68–70^. Recently, a method of generating SC states based on cavity electro-optic systems was also considered^71,72^. Moreover, continuous advances in electron quantum optics make it possible to think of solid-state quantum information processing based on flying qubits with controlled phases.

The purpose of the presented study is twofold; first, we show that the nonclassicality parameter in the form proposed by Kenfack and Życzkowski is related to the Rényi entropy for the Rényi index equal one half. Thereby, it allows us to interpret this quantity as an entropic measure of the area occupied by the negative part of the WDF in the phase space. Secondly, we investigate the influence of the relative phase encoded in the Schrödinger cat states on the dynamics of the WDF in the presence of the scattering center, assuming that the Gaussian wavepackets that form this bimodal state move in opposite directions. We quantitatively describe this dynamical problem in terms of the aforementioned fractional Rényi entropy.

## Theoretical framework

Here we introduce only indispensable facts concerning the phase-space formulation of the quantum theory which are directly used in this work. More details about this formulation can be found in the extensive literature related to this issue^14,49,73–78^. In the phase-space formulation of the quanta, the isolated one-particle quantum-mechanical system is characterized by the Weyl symbol of the Hamiltonian, ${\hat{H}}({\hat{x}},{\hat{p}})={\hat{p}}^2/(2m)+U({\hat{x}})$, which is given by the formula

1 $$\begin{aligned} H_W(x,p) = \int {dX}\; \left<x+\frac{X}{2}\right| {\hat{H}}({\hat{x}}, {\hat{p}}) \left| x-\frac{X}{2}\right> e^{-\frac{ipX}{\hbar }}. \end{aligned}$$

The Weyl symbol of the Hamiltonian for the considered system is equivalent to the classical Hamilton function, i.e. $H_W(x,p)=H(x,p) = p^2/(2m)+U(x)$, where *p* and *x* are the Weyl symbols of the momentum and position, respectively, and they are consistent with the classical counterparts; *U*(*x*) is the potential energy and *m* denotes the mass. In turn, the state of the considered quantum-mechanical system is represented by the WDF, which corresponds to the Weyl symbol of the density operator, ${\hat{\rho }}(t)$, rescaled by the factor $1/(2\pi \hbar )$. Especially for the state represented by such a density operator, the corresponding WDF can be expressed as follows,

2 $$\begin{aligned} \rho (x,p; t) = \frac{1}{2\pi \hbar } \int dX\left<x+\frac{X}{2}\right| {\hat{\rho }}(t) \left| x-\frac{X}{2}\right> e^{-\frac{ipX}{\hbar }}. \end{aligned}$$

Based on this expression, we conclude that the time evolution of the quantum system state in the phase space follows from the time dependence of the WDF. The equation of motion of this function for the isolated system was initially derived by Wigner in his original paper^1^. However, only further studies conducted by Moyal allowed introducing the structure called the ‘sine-Poisson’ bracket^2^. On the one hand, this discovery was an essential step in understanding the non-commutativity of the Poisson structure of the quantum phase space (p$\hbar$ase space), and on the other hand it allowed one to reformulate the Wigner’s original form of the equation of motion to the new form which is now called Moyal equation

3 $$\begin{aligned} i\hbar \partial _t\rho (x,p;t) = \left\{ H_W(x,p),\rho (x,p;t)\right\} _{\star }, \end{aligned}$$

where the symbol $\{\cdot ,\cdot \}_{\star }$ denotes the Moyal bracket of two phase-space functions^79^. Let us note that this expression can be reduced to the Poisson bracket in the classical limit, symbolically understood as $\hbar \rightarrow 0$. Consequently, the Moyal equation reduces to the Liouville equation, and quantumness is preserved only in the initial condition, while the movement is consistent with classical dynamics generated by the Liouville flow. It is also worth noting that the Moyal and Liouville dynamics are consistent in the case of the potential energy expressed by a polynomial of the order less than or equal to 2. However, for computational reasons, it is more convenient to formulate the equation of motion (3) slightly differently. Namely, using the Bopp shifts^80^, the Moyal equation can be transformed into the following form^52^.

4 $$\begin{aligned} \partial _t \rho (x,p;t) = -\frac{p}{m}\partial _x\rho (x,p;t) + \frac{1}{i\hbar } \bigg [ U\left( x+\frac{i\hbar }{2}\partial _p\right) - U\left( x-\frac{i\hbar }{2}\partial _p\right) \bigg ] \rho (x,p; t), \end{aligned}$$

where the first term in the RHS of this equation corresponds to the kinetic term, while the second term in the RHS represents the potential term which is inherently nonlocal^81^. The structure of this equation explicitly alludes to the Liouville equation, although the potential term has an intricate form. Nevertheless, finding the time evolution of the WDF requires specifying the initial condition for the Eq. (4) and solving the Cauchy problem for it.

After this nutshell foreword to the phase-space formulation of quanta, we now discuss one of the primary results of our work, namely the relationship between entropy and the nonclassicality parameter. We prove that for pure states the Rényi entropy for the Rényi index equal to one half can be considered the logarithmic measure of state nonclassicality. The assumption of the purity of the state is significantly important because it allows us to look at the WDF as the amplitude of the probability density in the phase space according to the arguments presented in Refs.^39,41^, i.e. the WDF represents the wave function on the phase space for the pure state being a particular solution of the Schrödinger equation written in the phase-space representation. On this basis, it can be concluded that the symplectically covariant squared modulus of the WDF is regarded as the probability density in the phase space, and the corresponding WDF norm with respect to the space $L^2({\mathbb {R}}^2)$ is given by the formula

5 $$\begin{aligned} ||{\rho }(x,p,t)||_{L^2({\mathbb {R}}^2)}= \left( \int _{{\mathbb {R}}^2}{dx dp}\; \left| \rho (x,p,t)\right| ^2 \right) ^{1/2} = \frac{1}{\sqrt{2\pi \hbar }}. \end{aligned}$$

Using this norm, along with the auxiliary function, ${\widetilde{\rho }}(x,p,t)$, introduced by Dias et al.^82^,

6 $$\begin{aligned} {\widetilde{\rho }}(x,p,t) = \frac{{\rho }(x,p,t)}{||{\rho }(x,p,t)||_{L^2({\mathbb {R}}^2)}}, \end{aligned}$$

to ensure correct normalization, we can express the Rényi entropy of the probability density, $|{\widetilde{\rho }}(x,p,t)|^2$, in the phase space for the Rényi index $\alpha$ in the form

7 $$\begin{aligned} S_{\alpha }(t) = \frac{1}{1-\alpha }\ln \left[ \int _{{\mathbb {R}}^2}{dx dp}\; \left( |{\widetilde{\rho }}(x,p,t)|^{2}\right) ^{\alpha }\right] . \end{aligned}$$

The obtained result can be regarded as an extension of Dias et al.’s result given by Eq. (22) in their work^82^ to the Rényi entropy. However, we are still restricted by the purity of the state represented by the WDF, which is interpreted as the wave function in the phase-space representation. On the other hand, taking into account that $|{\rho }(x,p,t)|=[{\rho }^2(x,p,t)]^{1/2}$, we get the result similar to the quantum Wigner–Rényi entropy defined for Wigner-positive states recently introduced by van Herstraeten and Cerf^47^. Moreover, this last result is also extended by Dias and Prata^48^ to the case of the arbitrary integrable Wigner functions associated with the Feichtinger states^83^.

After taking into consideration Eqs. (5) and (6), we can transform Eq. (7) into the form

8 $$\begin{aligned} S_{\alpha }(t) = \frac{1}{1-\alpha }\ln \left( \int _{{\mathbb {R}}^2} dx dp\; \left| {\rho }(x,p,t)\right| ^{2\alpha }\right) + \frac{\ln {\left( \sqrt{2\pi \hbar }\right) ^{2\alpha }}}{1-\alpha }. \end{aligned}$$

Let us note that the Wigner–Rényi entropy expressed by Eq. (8) for the fixed Rényi index $\alpha$ refers to the probability density in the phase space defined by Eq. (6). In the case of the Rényi index $\alpha =1/2$, the Wigner–Rényi entropy (8) can be algebraically reduced to the form

9 $$\begin{aligned} S_{1/2}(t) = 2\ln \left( \int _{{\mathbb {R}}^2}{dx dp}\;|\rho (x,p;t)|\right) + \ln {(2\pi \hbar )}. \end{aligned}$$

This observation is an important result because it allows us to conclude that the Wigner–Rényi entropy for the Rényi index $\alpha =1/2$ can be regarded as the entropic measure based on the WDF with a clear physical interpretation. To see this, it is enough to use the definition of the nonclassicality parameter introduced by Kenfack and Życzkowski^43^, namely

10 $$\begin{aligned} \delta (t) = \int _{{\mathbb {R}}^2}{dx dp}\; \left| \rho (x,p, t)\right| -1. \end{aligned}$$

Then, comparison of Eqs. (9) and (10) leads to the following relation between these two quantities,

11 $$\begin{aligned} S_{1/2}(t) = 2\ln \left[ \delta (t)+1\right] + \ln {(2\pi \hbar )}. \end{aligned}$$

Hence, we can conclude that the entropy $S_{1/2}$ can be regarded as a logarithmic measure of the nonclassicality of the state that is represented by the WDF and which is understood as the wave function of the pure state in the phase space. On the other hand, the nonclassicality parameter can be expressed by the entropy, $S_{1/2}(t)$, according to the formula

12 $$\begin{aligned} \delta (t) = \frac{1}{\sqrt{2\pi \hbar }}\exp {\left[ \frac{S_{1/2}(t)}{2}\right] } - 1. \end{aligned}$$

This result clearly shows that the nonclassicality of the state can be measured by the Wigner–Rényi entropy of order $\alpha =1/2$. In particular, we can conclude that for any Wigner-positive states, i.e. $\delta (t)=0$, we always obtain the constant value of this entropy, namely $S_{1/2}(t)=\ln {(2\pi \hbar )}$ which represents the logarithm of the quantum cell volume in the phase space. Moreover, analyzing the formula for entropy $S_{1/2}$ as a function of the nonclassicality parameter $\delta$ in time, we can conclude that this quantity may be a nonmonotonic function, generally. This is because during the time evolution of the WDF in dispersion media, where quantum phenomena play the essential role, changes in the nonclassicality parameter are observed in the form of its initial increase and subsequent decrease to a specific value. We often come across such situations in transport processes.

Let us return to the Wigner–Rényi entropy given by Eq. (8). This expression can be used to discuss the entropic uncertainty relation in the phase space^46,50,84–87^. A natural question arising from this issue concerns the lower bound of the Wigner–Rényi entropy with the Rényi index $\alpha \geqslant 1/2$. For the study of this problem, we use the results of Lieb’s work^88^, where the author proved that the lower bound for the $L^q$-norm of the WDF with $1 \leqslant q < 2$ and the upper bound with $q>2$, are saturated only by the Gaussian states. Let us define the function $I^{2\alpha }(t)$ for $\alpha >0$ in such a way that it corresponds to the $L^q$-norm considered in Lieb’s work, namely,

13 $$\begin{aligned} I^{2\alpha }(t) = 2^{1-\alpha }(\pi \hbar )^{\alpha -1}\int _{{\mathbb {R}}^2} {dx dp}\; |{\widetilde{\rho }}(x,p,t)|^{2\alpha }. \end{aligned}$$

Following the result of Theorem 1 from Lieb’s work^88^, we can find the upper bound of the integral (13) for $\alpha > 1$. Let us note that taking the natural logarithm scaled with $2^{\alpha -1}(\pi \hbar )^{1-\alpha }$ and then rescaling it with $1/(1-\alpha )$, we reconstruct the expression for the Wigner–Rényi entropy given by Eq. (8). Because for $\alpha > 1$, the term $1/(1-\alpha )$ in Eq. (8) is negative, and the logarithm is a monotonically increased function, the inequality from Theorem 1 changes sign, resulting in the lower, not upper bound for the Wigner–Rényi entropy for $\alpha > 1$. Referring to Theorem 2 of Lieb’s work^88^, we arrive at the lower bound for $1/2 \leqslant \alpha <1$. Now, we follow the same reasoning as in the previous proof for $\alpha > 1$. This result gives us a complete lower boundary for the Wigner–Rényi entropy with $\alpha \geqslant 1/2$. The boundary can be expressed in the form of the following inequality

14 $$\begin{aligned} S_{\alpha } (t) \geqslant \frac{1}{1-\alpha } \ln \left[ \frac{1}{\alpha } \left( \frac{2}{\pi \hbar }\right) ^{\alpha -1} \right] , \end{aligned}$$

which according to Lieb saturates iff $S_{\alpha } (t)$ is calculated for the Gaussian WDF. Moreover, we note that for $\alpha =1/2$, we reproduce our previous result, i.e. $S_{1/2}=\ln {2\pi \hbar }$, but with some qualitative difference, namely, we obtained this result for the class of the Wigner-positive states ($\delta =0$) of which the Gaussian states are a particular case.

Finally, let us note that the formula given by Eq. (14) extends the results of Dias et al.^82^ to the case $\alpha \geqslant 1/2$. However, this extension is a succession of the used interpretation of the WDF. Secondly, the authors only consider the Shannon entropy corresponding to Rényi index $\alpha =1$.

At the end of this discussion it is worth pointing out that Dias and Prata^48^ proposed the lower bound of Rényi-Wigner entropy of $|\rho (x,p,t)|$ for two separate cases of Rényi index, namely $\alpha \in (1,2)$ and $\alpha \geqslant 2$. In the same work, they proved the van Herstraeten-Cerf conjecture of the Wigner-positive states for $\alpha \geqslant 2$.

## Computational method

The general solution of the Moyal equation (4) can be written in the exponential form, such that

15 $$\begin{aligned} \rho (x,p;t) = {{\hat{\mathscr{U}}}}(t)\rho (x,p;0), \end{aligned}$$

where ${{\hat{\mathscr{U}}}}(t)$ is the time evolution operator. For the Moyal equation, the operator ${{\hat{\mathscr{U}}}}(t)$ is given by the following formula

16 $$\begin{aligned} {{\hat{\mathscr{U}}}}(t_i-t_0) = \exp \left[ -\frac{i}{\hbar }\left( {\hat{T}}+{\hat{U}}\right) (t_i-t_0) \right] , \end{aligned}$$

where ${\hat{T}}=-i\hbar p\partial _x/m$ and ${\hat{U}}=U\left( x+i\hbar \partial _p/2\right) -U\left( x-i\hbar \partial _p/2\right)$ are kinetic and potential operators, respectively.

In the numerical calculations, the solution can be obtained by acting on WDF repeatedly with the operator ${{\hat{\mathscr{U}}}}(\Delta t)$, where $\Delta t = 10$ a.u. is the time increment. Calculation-efficient form of the time evolution operator can be derived by applying the symmetric Strang splitting formula^89–91^

17 $$\begin{aligned} {{\hat{\mathscr{U}}}}(\Delta t) = \exp \left( -\frac{i}{2\hbar }{\hat{T}}\Delta t\right) \exp \left( -\frac{i}{\hbar }{\hat{U}}\Delta t\right) \exp \left( -\frac{i}{2\hbar }{\hat{T}}\Delta t\right) + O\left( \Delta t^3\right) . \end{aligned}$$

Using the partial Fourier transforms in the first or second variable defined as follows,

18 $$\begin{aligned} {\mathscr {F}}_{1,x\rightarrow \lambda }\rho (x,p; t) = \frac{1}{\sqrt{2\pi \hbar }}\int _{{\mathbb {R}}}dx\; e^{-\frac{ix\lambda }{\hbar }} \rho (x,p; t), \end{aligned}$$

19 $$\begin{aligned} {\mathscr {F}}^{-1}_{1,\lambda \rightarrow x}{\tilde{\rho }}(\lambda ,p; t) = \frac{1}{\sqrt{2\pi \hbar }}\int _{{\mathbb {R}}}d\lambda \;e^{\frac{ix\lambda }{\hbar }} {\tilde{\rho }}(\lambda ,p; t), \end{aligned}$$

20 $$\begin{aligned} {\mathscr {F}}_{2,p\rightarrow y}\rho (x,p; t) = \frac{1}{\sqrt{2\pi \hbar }}\int _{{\mathbb {R}}}dp\;e^{-\frac{ipy}{\hbar }} \rho (x,p; t), \end{aligned}$$

21 $$\begin{aligned} {\mathscr {F}}^{-1}_{2,y \rightarrow p}{\tilde{\rho }}(x,y; t) = \frac{1}{\sqrt{2\pi \hbar }}\int _{{\mathbb {R}}}dy\;e^{\frac{ipy}{\hbar }}{\tilde{\rho }}(x,y;t), \end{aligned}$$

one can obtain a formula for a single step of the time evolution of the WDF in the form

22 $$\begin{aligned} \rho (x,p;t_0+\Delta t) \approx {\mathscr {F}}^{-1}_{1,\lambda \rightarrow x} e^{-\frac{i\Delta t}{2m}\lambda p} {\mathscr {F}}_{1,x\rightarrow \lambda } {\mathscr {F}}^{-1}_{2,y\rightarrow p} e^{i\Delta t U_{\Delta }(x,y)} {\mathscr {F}}_{2,p\rightarrow y} {\mathscr {F}}^{-1}_{1,\lambda \rightarrow x} e^{-\frac{i\Delta t}{2m}\lambda p} {\mathscr {F}}_{1,x\rightarrow \lambda }\rho (x,p;t_0), \end{aligned}$$

where the auxiliary function $U_{\Delta }(x,y)$ is defined as the central difference of the potential energies, namely

23 $$\begin{aligned} U_{\Delta }(x,y) = U\left( x + \frac{y}{2}\right) - U\left( x - \frac{y}{2}\right) . \end{aligned}$$

Since Eq. (4) is defined for $x,p \in {\mathbb {R}}$, in order to perform numerical computation the phase space is limited to the box of size $[-L_x,L_x)\times [-L_p,L_p)$ with periodic boundary conditions imposed by the numerical method. Thus the used values of $L_x$ and $L_p$ are large enough to assure that the WDF vanishes in the vicinity of the boundary during the whole simulation. The computational box is discretized into the grid of size of $N_x \times N_p$ in the following way,

24 $$\begin{aligned} \begin{array}{cc} x_m = -L_x + m \Delta _x,\\ p_n = -L_p + n \Delta _p, \end{array} \end{aligned}$$

where $m \in \{0,1, \dots , N_x-1\}$, $n \in \{0,1, \dots , N_p-1\}$, and the steps on the computational grid are $\Delta _x = 2L_x/N_x$, $\Delta _p = 2L_p/N_p$. Numerical calculations were performed in atomic units ($\hbar =e=m=1$) and with the following parameters of the computational grid: $N_x = N_p = 1024$, $L_x = 1500$ a.u. and $L_p = 0.5$ a.u. For the efficient calculation of the Fourier transforms the Fast Fourier Transform (FFT) algorithm was used.

## Initial state

Numerical determination of the time evolution of the WDF from Eq. (22) requires establishing the initial condition. For this purpose, we assume the initial WDF in the form corresponding to the superposition of two Gaussians with the same widths, $\delta _x$, representing coherent states localized at different phase space points denoted by $(x_1, p_1)$ and $(x_2, p_2)$. According to this description, the general expression for this WDF can be written in the form

25 $$\begin{aligned} \begin{aligned} \rho (x,p)&= A^2 \frac{1-\beta }{\pi \hbar }\exp \left\{ -\frac{(x-x_1)^2}{2\delta _x^2} \right\} \exp \left\{ - \frac{2\delta ^2_x}{\hbar ^2}(p - p_1)^2 \right\} \\&\quad + A^2 \frac{\beta }{\pi \hbar }\exp \left\{ -\frac{(x-x_2)^2}{2\delta _x^2} \right\} \exp \left\{ - \frac{2\delta ^2_x}{\hbar ^2}(p - p_2)^2 \right\} \\&\quad + 2A^2 \frac{\sqrt{\beta (1-\beta )}}{\pi \hbar } \cos \left[ \theta +\frac{p_1-p_2}{\hbar }x+\frac{x_2-x_1}{\hbar }\left( p-\frac{p_1+p_2}{2}\right) \right] \\&\quad \times \exp \left\{ -\frac{1}{2\delta _x^2}\left( x-\frac{x_1+x_2}{2}\right) ^2 \right\} \exp \left\{ -\frac{2\delta _x^2}{\hbar ^2}\left( p-\frac{p_1+p_2}{2}\right) ^2 \right\} , \end{aligned} \end{aligned}$$

where parameter $\beta$ controls the amplitude ratio of the states, and $\theta$ is the relative phase between them. Besides this, the normalization factor *A* equals

26 $$\begin{aligned} A = \left[ 1+2 \sqrt{\beta (1-\beta )} \exp \left\{ -\frac{\delta _x^2}{\hbar ^2}(p_1-p_2)^2\right\} \exp \left\{ -\frac{1}{8\delta _x^2}(x_1-x_2)^2\right\} \cos \left[ \theta +\frac{(x_1+x_2)(p_1-p_2)}{2\hbar }\right] \right] ^{-1/2}. \end{aligned}$$

This form of the initial condition can be regarded as a generalization of the result presented in Ref.^52^ for the coherent superposition, and we refer to this as the Schrödinger cat state^10^ (SC state). For further investigation, we assume that both Gaussians move in the dispersive medium in opposite directions with the same value of the initial momentum, i.e. $p_2=-p_1$. On account of that, we can simplify expression (26) to the following form

27 $$\begin{aligned} \begin{aligned} \rho (x,p)&= A^2 \frac{1-\beta }{\pi \hbar }\exp \left\{ -\frac{(x-x_1)^2}{2\delta _x^2} \right\} \exp \left\{ - \frac{2\delta ^2_x}{\hbar ^2}(p - p_1)^2 \right\} \\&\quad + A^2 \frac{\beta }{\pi \hbar }\exp \left\{ -\frac{(x-x_2)^2}{2\delta _x^2} \right\} \exp \left\{ - \frac{2\delta ^2_x}{\hbar ^2}(p + p_1)^2 \right\} \\&\quad + 2A^2 \frac{\sqrt{\beta (1-\beta )}}{\pi \hbar } \cos \left[ \theta +\frac{2p_1}{\hbar }x+\frac{x_2-x_1}{\hbar }p\right] \exp \left\{ -\frac{1}{2\delta _x^2}\left( x-\frac{x_1+x_2}{2}\right) ^2 \right\} \exp \left\{ -\frac{2\delta _x^2}{\hbar ^2}p^2 \right\} , \end{aligned} \end{aligned}$$

with the normalization factor *A* given by the formula

28 $$\begin{aligned} A = \left[ 1+2 \sqrt{\beta (1-\beta )} \exp \left\{ -\frac{\delta _x^2}{\hbar ^2}2p_1^2\right\} \exp \left\{ -\frac{1}{8\delta _x^2}(x_1-x_2)^2\right\} \cos \left[ \theta +\frac{2p_1(x_1+x_2)}{2\hbar }\right] \right] ^{-1/2}. \end{aligned}$$

Referring to the previous study^52^, we assume the following values of the parameters which characterize this initial condition, namely $\beta = 0.5$, $\delta ^2_x = 500$ a.u., $x_1 = -300$, $x_2 = 300$ and $p_1 = 0.15$ a.u. Owing to this selection of the parameters, the presented initial condition (27) is the phase-space representation of the SC state given by the superposition of two well-separated and well-localized Gaussians approaching each other with the same momenta. Let us note that the relative phase still remains to be a free parameter of the initial condition, creating the possibility of researching its influence on the WDF dynamics generated by solving the Moyal equation in inhomogeneous dispersive media. Of course, the problem formulated in this way is too general; hence we decided to conduct a study on the three-element class of the considered bimodal states, which are well recognized in the literature. The class of these states consists of the odd and even SC states for which the relative phases are $\theta =0$ and $\theta =\pi$, respectively, and the Yurke-Stoler state with $\theta =\pi /2$. The influence of $\theta$ on the value of the normalization factor given by Eq. (28) has been found to be negligibly small due to the numerical values of the prefactors $\exp \left( -2p_1^2\delta _x^2/\hbar ^2\right)$ (approximately $1.7 \times 10^{-10}$ [a.u.]) and $\exp \left( -{(x_1-x_2)^2}/{(8\delta _x^2)}\right)$ (approximately $8.2 \times 10^{-40}$ [a.u.]).

## Results and discussion

**Figure 1:**
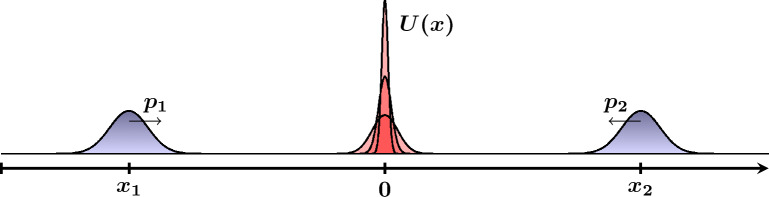
Conceptual sketch of the Schrödinger cat state interacting with the repulsive potential barrier with varying parameters.

We now turn to the results of the numerical simulation, in which we investigate the dynamics of the three-element family of SC states described by the WDF in the form given by Eq. (27), which move in a dispersive medium with the repulsive potential barrier. The barrier has the form of the Gaussian function,

29 $$\begin{aligned} U(x) = U_0\exp \left[ -\frac{(x-X_B)^2}{w^2} \right] , \end{aligned}$$

where $U_0$ is the strength of the barrier having width *w*, centered at $X_B$. The barrier is assumed to be located in the center of the simulation region ($X_B = 0$) whereas the remaining parameters take on the following values: $U_0 = 0.008$ a.u., $w^2 = 50$ a.u.; we will refer to this set of parameters as the standard parameters. An explanatory figure of the proposed setup is presented in Fig. 1.

We solved the Moyal equation (4) numerically by applying the second-order split-operator method according to Eq. (17).

**Figure 2:**
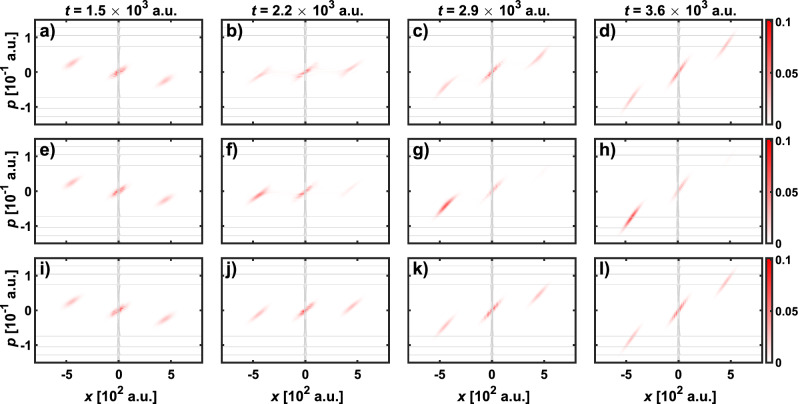
The phase space snapshots of the probability density on the phase space, $|\rho (x,p,t)|^2$, for the even SC state (first row), YS state (second row) and odd SC state (third row) at different times during interaction with the barrier in the form of a Gaussian potential. The equipotential lines of the classical Hamiltonian of the system under consideration are indicated by the grey contour lines.

The resulting time evolution of the probability density on the phase space, $|\rho (x,p,t)|^2$, is presented in Fig. 2 containing snapshots at $t=1.5$, 2.2, 2.9 and $3.6\times 10^3$ a.u. At these time instants, the probability density on the phase space occupies mostly that part of the space in the neighbourhood of the potential barrier, and the dynamics of the state is therefore determined by the characteristics of the potential. Earlier, that is before the situation illustrated in Fig. 2, the state evolves freely (details of the dynamics in terms of the WDF are presented in the Supplementary Information S1). When the interaction with the barrier starts, all three states retain their original symmetry, as visible in Fig. 2a,e,i. Then, for the assumed standard parameters of the barrier, interaction with the barrier takes place in the above-barrier regime resulting in changes of the probability density on the phase space depending on the chosen initial state. For the SC states, both odd (Fig. 2b–c) and even (Fig. 2j–k), interaction with the barrier does not break the symmetry of the initial state. In the case of the odd SC state, the fringes visible in Fig. 2b are the result of quantum interference. A similar phenomenon occurs for the even SC state; due to the small amplitude, it is not visible in Fig. 2j, but snapshots of the even SC state showing this phenomenon are available in the Supplementary Information S1. The situation is considerably different for the YS state interacting with the barrier in the above-barrier regime. As shown in Fig. 2f–g, such interaction of the YS state with a symmetric potential barrier leads to asymmetry of the initial state resulting in most of the probability density on the phase space remaining to the left of the barrier. Evolution of the YS state produces interference fringes visible in Fig. 2f. After interaction with the barrier, the free evolution takes place again, as shown in Fig. 2d,h,l. The SC states remain symmetrical, while YS states stay asymmetric. Although the time evolution of the even and odd SC states has the same effect in terms of keeping the symmetry of the initial state, the interaction of these states with the barrier proceeds differently. It is worth noting that although during the interaction with the barrier the even and odd SC states preserve the initial symmetry of the probability density on the phase space, the dynamics of the interaction is different.

**Figure 3:**
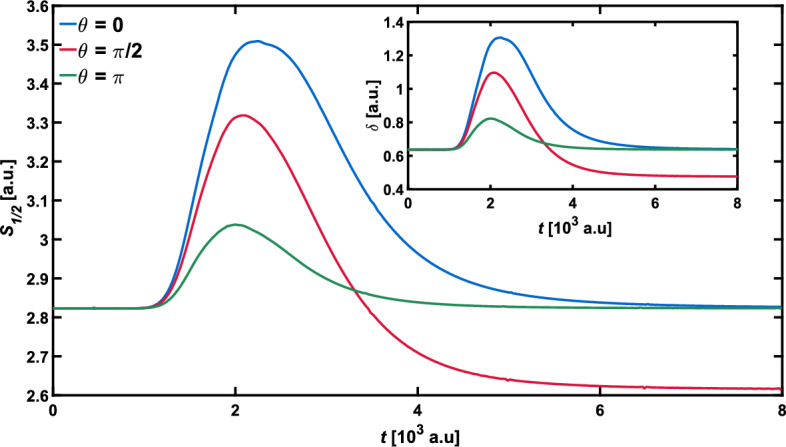
Influence of the relative phase $\theta$ on the Wigner–Rényi entropy of order $\alpha = 1/2$, for the standard parameters of the barrier. Inset shows the nonclassicality parameter $\delta$.

That difference can be identified with help of two quantities, the nonclassicality parameter $\delta$ and the Wigner–Rényi entropy of order $\alpha = 1/2$. Both are shown in Fig. 3 for the even and odd SC states and the Yurke–Stoler state. As visible in Fig. 3, the Wigner–Rényi entropy and the nonclassicality parameter change almost in the same manner for each considered case, which is in accordance with the derived relation (12) linking $\delta$ and $S_{1/2}$. Both of those dynamical characteristics, $S_{1/2}$ and $\delta$, of the odd SC state ($\theta = 0$) have maximal values larger than in the case of the even SC state ($\theta = \pi$). In addition, the characteristics of the even SC state approach the final constant value much faster.

The system was investigated for varying values of the parameters $U_0$ and $w^2$ to find out how this asymmetry depends on the strength and width of the barrier. First, for the fixed standard $w^2=50$ a.u. the strength of the barrier was varied in the range of $U_0 \in \{0.004, 0.008, \dots , 0.02 \}$. Then, for the standard $U_0 = 0.008$ a.u. the barrier width was varied in the range of $w^2 \in \{50, 148, 216, 298, 500\}$. The investigated values of $w^2$ correspond to uniform changes of the barrier width *w*.

**Figure 4:**
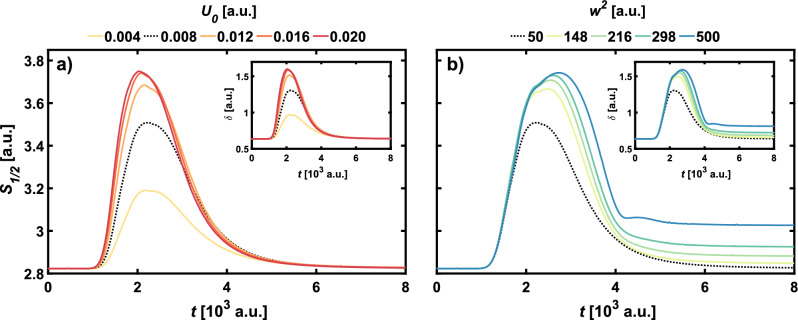
Influence of (**a**) parameter $U_0$ and (**b**) parameter $w^2$ on the Wigner–Rényi entropy $S_{1/2}$ for the even Schrödinger Cat WDF ($\theta = 0$). The inset shows the nonclassicality parameter $\delta$. Thicker, black dashed line indicates results for the standard parameters of the barrier, namely $U_0 = 0.008$ a.u. and $w^2 = 50$ a.u.

**Figure 5:**
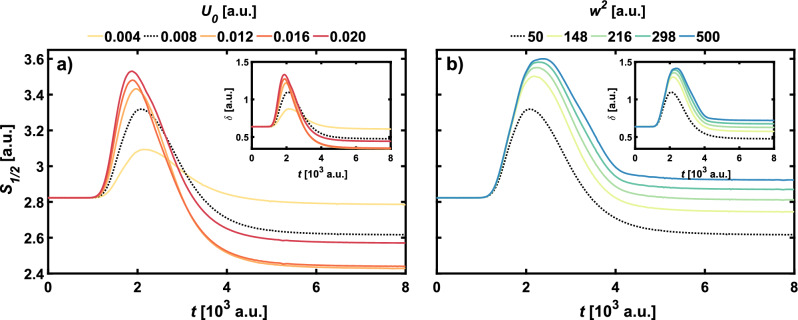
Influence of (**a**) parameter $U_0$ and (**b**) parameter $w^2$ on the Wigner–Rényi entropy $S_{1/2}$ for the Yurke–Stoler WDF ($\theta = \pi /2$). The inset shows the nonclassicality parameter $\delta$. Thicker, black dashed line indicates results for the standard parameters of the barrier, namely $U_0 = 0.008$ a.u. and $w^2 = 50$ a.u.

**Figure 6:**
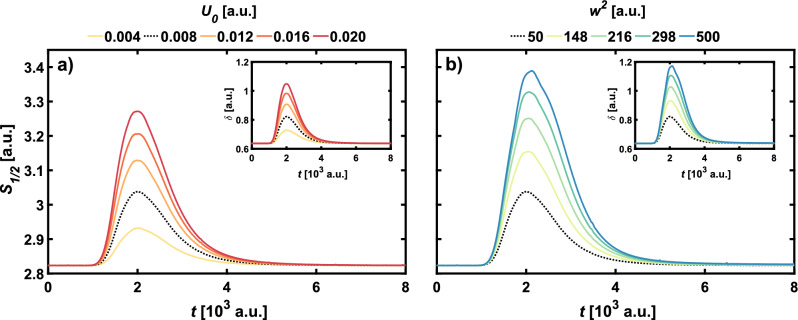
Influence of (**a**) parameter $U_0$ and (**b**) parameter $w^2$ on the Wigner–Rényi entropy $S_{1/2}$ for the odd Schrödinger Cat WDF ($\theta = \pi$). The inset shows the nonclassicality parameter $\delta$. Black dashed line indicates results for the standard parameters of the barrier, namely $U_0 = 0.008$ a.u. and $w^2 = 50$ a.u.

Figures 4 and 6 show the influence of the width and height of the barrier on the dynamical characteristics of the odd and even SC states. The nonclassicality parameter and the Wigner–Rényi entropy $S_{1/2}$ are also presented for the Yurke-Stoler state (Fig. 5) for various sizes of the barrier. In all figures, bold, dashed black lines indicate the Wigner–Rényi entropy for the standard barrier parameters that correspond to interactions in the above-barrier regime. The results presented in Fig. 4 show that for the even SC state, if barriers are higher than $U_0 = 0.012$ a.u. and wider than $w^2 = 148$ a.u., the peak values of the Wigner–Rényi entropy is the largest among the considered states, which also corresponds to the high nonclassicality expressed by the nonclassicality parameter $\delta$. For barrier height equal $U_0 = 0.004$ a.u. the entropy not only has the lowest maximal value, but also it is the fastest to reach the constant final value. While in the case of variable barrier height, shown in Fig. 4a, the entropy and nonclassicality parameter take on the final value equal to the initial one, the situation changes for variable barrier width. As shown in Fig. 4b, only for the standard parameters of the barrier the initial and final values of $S_{1/2}$ and $\delta$ are the same. With an increase in the width of the barrier, the final values are rising.

This allows us to conclude that increasing the width of the potential barrier results in an increasingly nonclassical state at the end of the simulation. It should also be noted that, in contrast to the change in height, the wider the barrier, the faster the entropy and nonclassicality parameters approach the final constant value. In the case of the YS state, it can already be observed for a barrier with standard parameters that the final value of the entropy and the nonclassicality parameter is different—smaller—than the initial value. This is related to the asymmetrization of the initial state. As can be seen in Fig. 5 lowering the barrier to $U_0=0.004$ a.u. results in bringing the final value closer to the initial value, leading to the conclusion that lowering the barrier has a positive effect on maintaining the initial symmetry of the YS state. Increasing the barrier height results in a larger asymmetry, up to $U_0=0.02$ a.u. where the trend reverses, and the final values of the entropy and nonclassicity parameter are close to the final values obtained for the standard barrier parameters. A similar effect on the symmetry of the final state can be observed when the barrier width increases. This means that the strength of asymmetrization can be modified by controlling the potential parameters.

Figure 6 shows the effect of changes in the potential parameters on the Wigner–Rényi entropy and the nonclassicality parameter of the odd SC state. Although the effect of the barrier height $U_0$ is similar to that for the case of the even SC state shown in Fig. 4, it should be noted that for all of the considered barrier heights the entropy reaches the lowest maximal values among the considered states. This means that the evolution of the odd SC state is less nonclassical than for the even SC state. Unlike the even SC state, in the case of changes in the width of the potential barrier for the odd SC state, there is no change in the final value of $S_{1/2}$ and $\delta$ relative to the initial value. Regardless of the parameters of the potential, the initial symmetry of the odd SC state will be always preserved after interaction with the barrier. As for the even SC state, we observe a faster flattening for lower $U_0$, but the same trend is also preserved for $w^2$ changes. In contrast to the even SC state, in the case under consideration the wider the potential barrier, the later the Wigner–Rényi entropy and the nonclassicality parameter flatten out.

## Concluding remarks

The phase-space formulation of quantum theory based on the Wigner distribution function allows us to take an alternative view of the quantitative description of the dynamical aspect of quantum systems. Using this approach, we have analyzed the dynamics of the three-element family of Schrödinger cat states in the phase space. The distinction between members of this family is based on the adoption of the established values of the relative phase encoded in the general form of the Schrödinger cat state. In the present studies, we have focused on a quantitative description of dynamic changes in the nonclassicality of the considered states during their interaction with the repulsive barrier in terms of the fractional Wigner–Rényi entropic measure. The results obtained by us can be divided into two parts.

In the first part of the presented studies, we have introduced the Wigner–Rényi entropy using the square of the modulus of the Wigner function. This quantity is interpreted as the probability density in the phase space only for pure states. Due to this observation, we have found a relationship between the Wigner–Rényi entropy for the index Rényi $\alpha =1/2$ and the nonclassicality parameter introduced by Kenfack and Życzkowski, which is regarded as a measure of the area in the phase space occupied by the negative part of the Wigner function, and which is also considered as an indicator of quantumness. Furthermore, we have found a lower bound for the Wigner–Rényi entropy generated by the modulus squared of the Wigner function.

In the second part of our studies, the previously introduced concepts have been used to study the dynamics of the three-element family of Schrödinger cat states consisting of the odd, even and Yurke-Stoler states, with a scattering center in the form of the Gaussian barrier. At the initial moment, the considered family of quantum states has been modeled by a coherent superposition of two wave packets located on opposite sides of the barrier with appropriately selected relative phases in such a way as to reproduce the above-mentioned family. In our studies, we have used the wave packets having equal but oppositely directed momenta, moving towards a scattering barrier placed exactly in the middle of the distance between the packets. As a result of the performed simulations, we have noticed that independently of the choice of the relative phase, the Wigner–Rényi entropy measuring the nonclassicality of the considered states in the region of their interaction with the barrier changes significantly. Simultaneously, we have observed that the degree of nonclassicality for the even and odd Schrödinger cat states is preserved, while for the Yurke-Stoler states, we have observed a decrease of the degree of nonclassicality with respect to the initial moment. The obtained result is related to the emergence of an asymmetry in the interaction of packets forming the Yurke-Stoler state with the Gaussian barrier.

We have carried out calculations in two energy regimes, i.e. we have considered the case of scattering of states over the barrier and their tunneling. The transition between these regimes has been possible by controlling the height of the barrier while maintaining all the parameters of the Schrödinger cat states. As a result, we have observed that the considered fractional Wigner–Rényi entropy has the same value before the odd and even states enter the region of interaction with the potential barrier and after they leave. In contrast, for the Yurke-Stoler states, we have not observed any change during scattering over the barrier, while during the tunneling the Wigner–Rényi entropy decreases from its initial value. In addition, we have also investigated the influence of the Gaussian barrier width on the dynamic changes of the fractional Wigner–Rényi entropy. In this case, we have noticed that increasing the barrier width causes an increase in the Wigner–Rényi entropy for all the considered states during their interaction with the barrier. In contrast, after leaving this area, the even and Yurke-Stoler states are characterized by greater fractional Wigner–Rényi entropy compared to the initial value. Nevertheless, we do not observe such a change in the odd state’s fractional Wigner–Rényi entropy.

In conclusion, the main objective of the presented studies has been to show that the Wigner–Rényi entropy for the Rényi index equal one half can be interpreted as the logarithmic measure of the area occupied by the negative part of the Wigner function in the phase space. The obtained result is significant because it shows the direct relationship between the considered entropy and the nonclassicality parameter of Kenfack and Życzkowski^43^. We have demonstrated that this fractional entropy can be interpreted in terms of the quantumness of states expressed by the negativity of the Wigner function. At the same time, we have found the lower bound of the Wigner–Rényi entropy defined for the probability density distribution in the phase space understood as the squared module of the Wigner function representing the pure state. In the limiting case of the Rényi index equal one the result is consistent with the known lower bound for the Shannon entropy for pure states. The results obtained from the numerical simulations prove the validity of the introduced tool, and we expect that it will be an introduction to further research on fractional Wigner–Rényi entropies for pure states.

### Supplementary Information

Supplementary Information.
