# Supplementary material for: Dynamical entropic measure of nonclassicality of phase-dependent family of Schrödinger cat states
Source: Sci Rep. 2023 Sep 27;13:16266. doi: 10.1038/s41598-023-43421-2 (PMC10533523; doi:10.1038/s41598-023-43421-2)
Supplement: Supplementary file 1 — Supplementary Information. [file 41598_2023_43421_MOESM1_ESM.pdf]

Supplementary Information: Dynamical entropic measure of nonclassicality of phase-dependent family of Schrödinger cat states

M. Kalka[1], B. J. Spisak[1,*], D. Woźniak[1], M. Wołoszyn[1], and D. Kołaczek[2]

[1]AGH University of Krakow, Faculty of Physics and Applied Computer Science, al. Mickiewicza 30, 30-059 Krakow, Poland
[2]University of Agriculture in Kraków, Department of Applied Mathematics, ul. Balicka 253c, 30–198 Kraków, Poland
[*]email: bjs@agh.edu.pl

Note on the time evolution of the Wigner distribution function

In the space-phase formulation of quantum theory, the state of a quantum mechanical system is represented by a state function defined on the phase space. This space is generated by canonically coupled variables, which are momentum and position. One way to realize such a description is to choose a Wigner distribution function (WDF) which, due to its properties, is considered a canonical example of a nonclassical probability density distribution function.
The WDF for the pure state represented by the normalized wavefunction $\psi \in L^2(\mathbb{R})$ is given by the formula

$$\rho(x,p;t) = \frac{1}{2\pi\hbar}\int dX\,\psi^*\left(x+\frac{X}{2}\right)\psi\left(x-\frac{X}{2}\right)e^{-\frac{ipX}{\hbar}}. \tag{1}$$

With the distribution function defined above, its zeroth moments with respect to position and momentum can be written as

$$n(x;t) = \int dp\,\rho(x,p;t), \tag{2}$$

and

$$\widetilde{n}(p;t) = \int dx\,\rho(x,p;t). \tag{3}$$

The functions $n(x;t)$ and $\widetilde{n}(p;t)$ correspond to the well-defined marginal distributions, interpreted as the probability densities in real and momentum space, respectively, thus overcoming the difficulty arising from the fact that the WDF may assume negative values, which is a fundamental obstacle to interpret it as a probability distribution function in the Kolmogorov sense.
The time evolution of the WDF can be determined by solving the Cauchy problem for the Moyal equation,

$$\partial_t\rho(x,p;t) = -\frac{p}{m}\partial_x\rho(x,p;t) + \frac{1}{i\hbar}\left[U\left(x+\frac{i\hbar}{2}\partial_p\right) - U\left(x-\frac{i\hbar}{2}\partial_p\right)\right]\rho(x,p;t), \tag{4}$$

using the initial condition in the form

$$\begin{aligned}\rho(x,p;t=0) = {} & A^2\frac{1-\beta}{\pi\hbar}\exp\left\{-\frac{(x-x_1)^2}{2\delta_x^2}\right\}\exp\left\{-\frac{2\delta_x^2}{\hbar^2}(p-p_1)^2\right\} \\ & + A^2\frac{\beta}{\pi\hbar}\exp\left\{-\frac{(x-x_2)^2}{2\delta_x^2}\right\}\exp\left\{-\frac{2\delta_x^2}{\hbar^2}(p+p_1)^2\right\} \\ & + 2A^2\frac{\sqrt{\beta(1-\beta)}}{\pi\hbar}\cos\left[\theta+\frac{2p_1}{\hbar}x+\frac{x_2-x_1}{\hbar}p\right]\exp\left\{-\frac{1}{2\delta_x^2}\left(x-\frac{x_1+x_2}{2}\right)^2\right\}\exp\left\{-\frac{2\delta_x^2}{\hbar^2}p^2\right\},\end{aligned} \tag{5}$$

with the normalization factor A given by the formula

$$A = \left[1+2\sqrt{\beta(1-\beta)}\exp\left\{-\frac{\delta_x^2}{\hbar^2}2p_1^2\right\}\exp\left\{-\frac{1}{8\delta_x^2}(x_1-x_2)^2\right\}\cos\left[\theta+\frac{2p_1(x_1+x_2)}{2\hbar}\right]\right]^{-1/2}. \tag{6}$$

The WDF used as the initial condition at $t = 0$ results from the wavefunction describing the linear superposition of two states, given by

$$\psi(x,t=0) = A\sqrt[4]{\frac{1}{2\pi\delta_x^2}}\left\{\sqrt{1-\beta}\exp\left[-\frac{(x-x_1)^2}{4\delta_x^2}+\frac{i}{\hbar}p_1x\right]+\sqrt{\beta}e^{i\theta}\exp\left[-\frac{(x-x_2)^2}{4\delta_x^2}-\frac{i}{\hbar}p_1x\right]\right\}. \tag{7}$$

This wavefunction corresponds to two Gaussian wavepackets with equal widths δ_x, the first centered at x_1 with initial momentum p_1, and the second centered at x_2 with initial momentum p_2. All numerical values of the parameters characterizing the initial state are assumed to be the same as in our previous work[1], i.e. $\beta = 0.5$, $\delta_x^2 = 500$ a.u., $x_1 = -300$, $x_2 = 300$, $p_1 = 0.15$ a.u., and $p_2 = -0.15$ a.u. Note that for the used initial condition, the explicit form of the marginal distributions can be determined, namely,

$$\begin{aligned} n(x) &= A^2\frac{\beta}{\sqrt{2\pi\delta_x^2}}\exp\left\{-\frac{(x-x_2)^2}{2\delta_x^2}\right\}+\frac{1-\beta}{\sqrt{2\pi\delta_x^2}}\exp\left\{-\frac{(x-x_1)^2}{2\delta_x^2}\right\} \\ &+ A^2\frac{\sqrt{2\beta(1-\beta)}}{\sqrt{\pi\delta_x^2}}\exp\left\{-\frac{1}{2\delta_x^2}\left(x-\frac{x_1+x_2}{2}\right)^2\right\}\exp\left\{-\frac{2x^2-2x(x_1+x_2)+x_1^2+x_2^2}{4\delta_x^2}\right\}\cos\left[\frac{2p_1}{\hbar}x+\theta\right], \end{aligned} \tag{8}$$

and

$$\begin{aligned} \tilde{n}(p) &= A^2\beta\sqrt{\frac{2}{\pi}}\frac{\sqrt{\delta_x^2}}{\hbar}\exp\left\{-\frac{2(p-p_2)^2\delta_x^2}{\hbar}\right\}+A^2(1-\beta)\sqrt{\frac{2}{\pi}}\frac{\sqrt{\delta_x^2}}{\hbar}\exp\left\{-\frac{2(p-p_1)^2\delta_x^2}{\hbar}\right\} \\ &+ 2A^2\Gamma\sqrt{\beta(1-\beta)}\sqrt{\frac{2}{\pi}}\frac{\sqrt{\delta_x^2}}{\hbar}\exp\left\{-\frac{2(p+p_1)^2\delta_x^2}{\hbar}\right\}\cos\left[\frac{p(x_2-x_1)}{\hbar}+\frac{p_1(x_1+x_2)}{\hbar}+\theta\right]. \end{aligned} \tag{9}$$

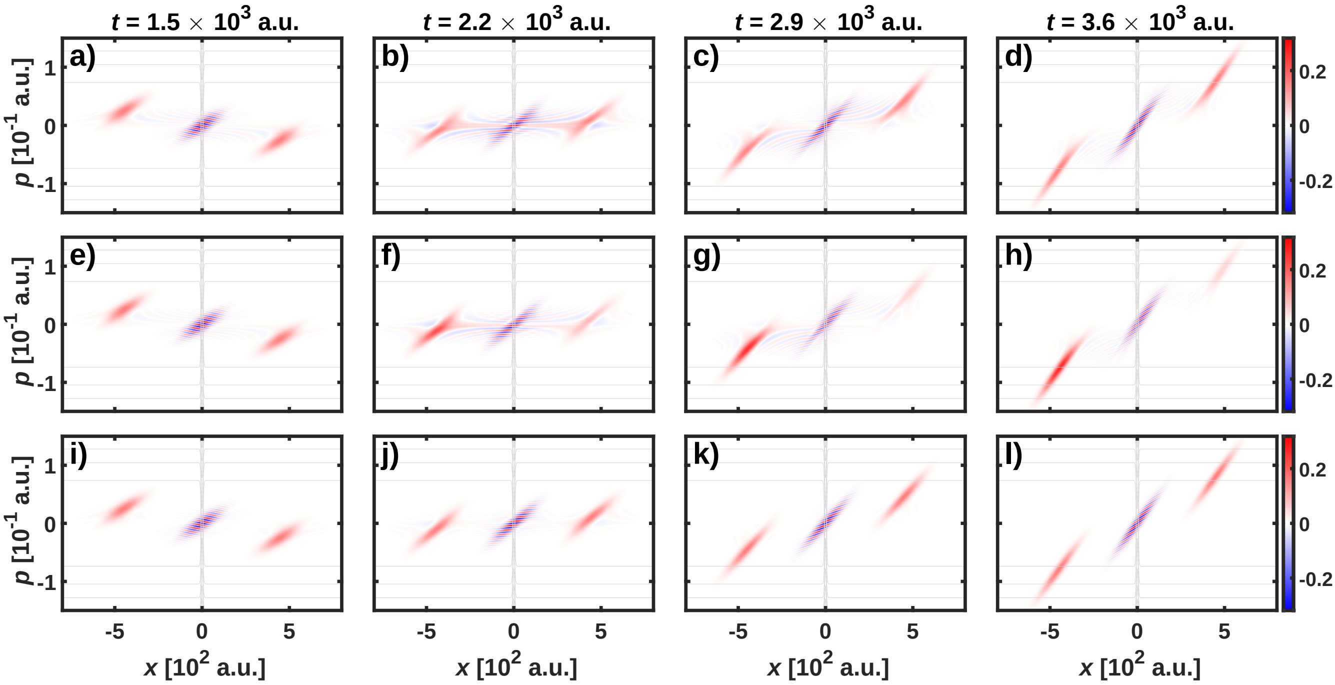

Figure 1. The phase space snapshots of the WDF of the even SC state (first row), YS state (second row) and odd SC state (third row) at different times during interaction with the barrier in the form of a repulsive Gaussian potential. The equipotential lines of the classical Hamiltonian of the system under consideration are indicated by the grey contour lines.

Depending on the choice of the phase θ, the function (7) describes the WDF of an even Schrödinger cat (SC) state ($\theta = 0$), odd SC state ($\theta = \pi$) or Yurke-Stoler (YS) state ($\theta = \pi/2$). As discussed in the paper, the phase *theta* barely influences the initial state. Furthermore, Eqs. (8) and (9) allow describing the influence of the θ parameter on the value of the interference term of the initial probability densities in both real and momentum spaces. The prefactors responsible for damping out the interference

parts of Eqs. (8) and (9) are of the order of 10^{-10} for the momenta distribution and 10^{-40} for the probability density in real space. This means that the impact of the phase on the marginal distributions at $t = 0$ is also negligible.

In order to determine the time evolution of the WDF for the system under consideration, the split-operator method[2,3] has been used to solve the Cauchy problem given above. In contrast to Fig. 2 in the main text, Fig. 1 included in this supplementary material shows the phase-space snapshots of the WDF, and not the module squared of the WDF. Of course, the dynamics of the WDF presented in Fig. 1 is qualitatively the same as the dynamics of the square of the module presented in the paper, but the WDF allows us to catch some nuances of the presented system. First of all, negative WDF values evolving in the phase space stand out in Fig. 1, which provides information about the nonclassicality of the studied state. Even before the interaction with the barrier starts, the negative values of the WDF are clearly visible for all three considered initial states [see Figs. 1 (a), (e), (i)]. This is the result of the interference term in Eq. (5), and hence all three states have non-classical characteristics. This conclusion is supported by the analysis of the two nonclassicality measures contained in the paper, the nonclassicality parameter δ and the entropic nonclassicality measure $S_{1/2}$. Another important feature of the studied system, highlighted by Fig. 1, is the process of asymmetrization of the YS state after interaction with the potential barrier. As can be seen in Figs. 1 (f), (g) and (h), the initially symmetric WDF for the YS state becomes asymmetric during interaction with the potential barrier and continues the free evolution in the asymmetric form. Phase-space snapshots of the WDF allow more pronounced interference patterns to be observed in phase space for the odd SC state, as shown in Figs. 1 (j) and (k).

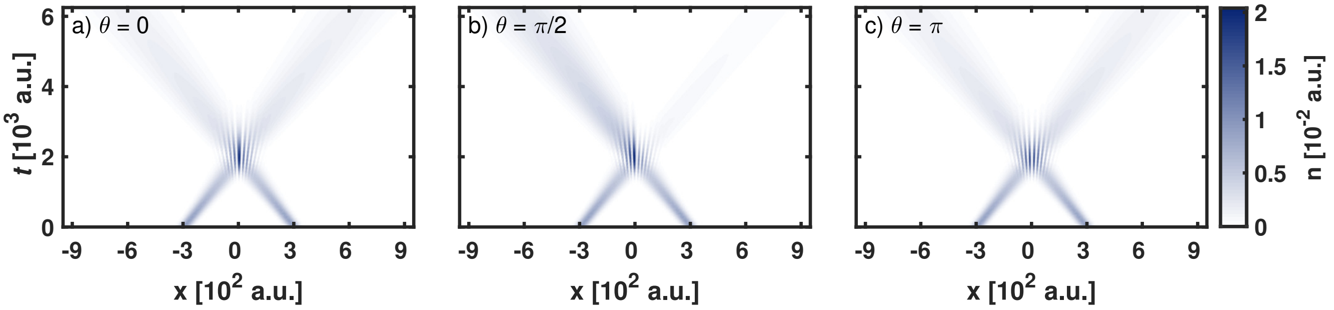

Figure 2. Influence of the relative phase θ on the probability density in real space $n(x,t)$, for the standard parameters of the barrier; (a) and (c) for the initial even and odd SC states respectively and (b) for the initial YS state.

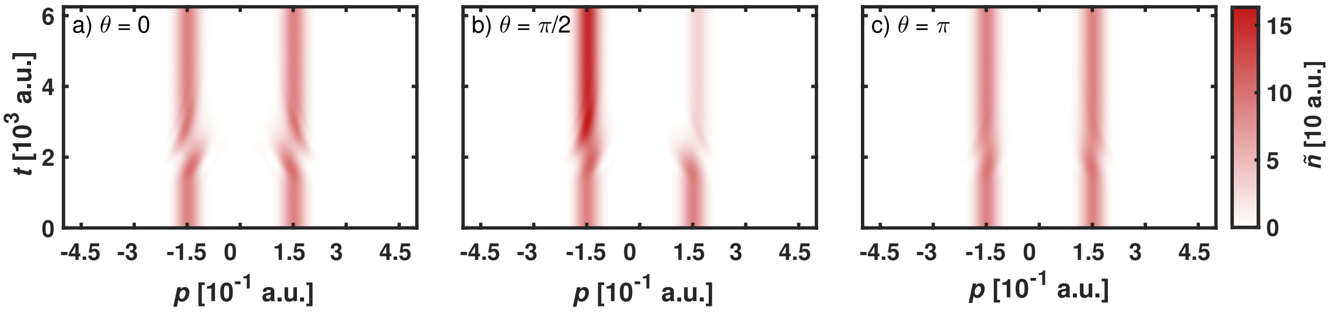

Figure 3. Influence of the relative phase θ on the probability density in momentum space $\tilde{n}(p,t)$, for the standard parameters of the barrier; (a) and (c) for the initial even and odd SC states respectively and (b) for the initial YS state.

In this supplementary material, we also present the time evolution of the marginal distributions, in space (Fig. 2) and momentum (Fig. 3), for all three considered types of states. Figs. 3 and 2 show how the momentum and position marginal distributions change during the simulations performed for the even SC state (Figs. 3 (a) and 2 (a)), odd SC state (Figs. 3 (b) and 2 (b)) and YS state (Figs. 3 (c) and 2 (c)). In empty space, the considered states are symmetrical; however, in the presence of the potential barrier, the symmetry of the state vanishes when the phase θ is modified. The largest asymmetry is observed in the case of the YS state: approximately 85% of WDF remains in the area to the left of the barrier after the interaction. In addition, Fig. 2 shows clear interference fringes visible in the position space during the interaction of each state with the potential barrier. Figs. 2 and 3 illustrate the free evolution of the WDF observed before and after interaction with the barrier.

References

1. Kołaczek, D., Spisak, B. J. & Wołoszyn, M. Phase-space studies of backscattering diffraction of defective Schrödinger cat states. *Sci. Reports* **11**, 11619, DOI: 10.1038/s41598-021-90738-x (2021).

2. Cabrera, R., Bondar, D. I., Jacobs, K. & Rabitz, H. A. Efficient method to generate time evolution of the Wigner function for open quantum systems. *Phys. Rev. A* **92**, 42122, DOI: 10.1103/PhysRevA.92.042122 (2015).

3. Kołaczek, D., Spisak, B. J. & Wołoszyn, M. The Phase–Space Approach to time Evolution of Quantum States in Confined Systems: the Spectral Split–Operator Method. *Int. J. Appl. Math. Comput. Sci.* **29**, 439, DOI: 10.2478/amcs-2019-0032 (2019).
